# Stepping to phase-perturbed metronome cues: multisensory advantage in movement synchrony but not correction

**DOI:** 10.3389/fnhum.2014.00724

**Published:** 2014-09-11

**Authors:** Rachel L. Wright, Laura C. Spurgeon, Mark T. Elliott

**Affiliations:** School of Psychology, College of Life and Environmental Sciences, University of BirminghamEdgbaston, Birmingham, UK

**Keywords:** sensorimotor synchronization, multisensory integration, movement synchronization, modality effects, timing, phase correction, stepping, gait

## Abstract

Humans can synchronize movements with auditory beats or rhythms without apparent effort. This ability to entrain to the beat is considered automatic, such that any perturbations are corrected for, even if the perturbation was not consciously noted. Temporal correction of upper limb (e.g., finger tapping) and lower limb (e.g., stepping) movements to a phase perturbed auditory beat usually results in individuals being back in phase after just a few beats. When a metronome is presented in more than one sensory modality, a multisensory advantage is observed, with reduced temporal variability in finger tapping movements compared to unimodal conditions. Here, we investigate synchronization of lower limb movements (stepping in place) to auditory, visual and combined auditory-visual (AV) metronome cues. In addition, we compare movement corrections to phase advance and phase delay perturbations in the metronome for the three sensory modality conditions. We hypothesized that, as with upper limb movements, there would be a multisensory advantage, with stepping variability being lowest in the bimodal condition. As such, we further expected correction to the phase perturbation to be quickest in the bimodal condition. Our results revealed lower variability in the asynchronies between foot strikes and the metronome beats in the bimodal condition, compared to unimodal conditions. However, while participants corrected substantially quicker to perturbations in auditory compared to visual metronomes, there was no multisensory advantage in the phase correction task—correction under the bimodal condition was almost identical to the auditory-only (AO) condition. On the whole, we noted that corrections in the stepping task were smaller than those previously reported for finger tapping studies. We conclude that temporal corrections are not only affected by the reliability of the sensory information, but also the complexity of the movement itself.

## Introduction

Rhythmic entrainment of movements to an auditory beat is a task we can achieve with little apparent effort. Neuroimaging studies have highlighted that simply listening to an auditory rhythm engages motor areas in the brain (Grahn and Brett, 2007). The human ability to dance (Bläsing et al., 2012) or play music within an ensemble (Wing et al., 2014) without drifting out of time is facilitated by continuous temporal corrections to movements (Vorberg and Wing, 1996; Vorberg and Schulze, 2002), which ensures we remain in synchrony with the beat. A linear phase correction process can describe the maintenance of synchrony with the beat where the temporal error, or asynchrony, between the time of the perceptual event and the motor response is corrected by an internal timekeeper. The timekeeper generates and continuously adjusts the intervals between motor responses based on a proportion of the preceding asynchrony (Vorberg and Wing, 1996; Vorberg and Schulze, 2002). Importantly, the temporal correction to motor responses can be quantified through the use of a phase-perturbation paradigm (Repp, 2001, 2002; Elliott et al., 2009a). This involves participants synchronizing to an isochronous metronome during which a beat is perturbed such that it occurs earlier or later than expected. Participants correct the resulting large timing error on subsequent motor responses, which can be quantified to provide a measure of correction. Hence, it is possible to quantify timing accuracy of motor responses through a number of measures including asynchrony, interval production and temporal corrections.

The automaticity of being able to synchronize to an auditory beat does not transfer to other modalities. In particular, auditory metronome cues predominate over discrete visual cues in synchronization tasks involving finger tapping (Repp and Penel, 2002, 2004; Kato and Konishi, 2006). Subsequently, attempting to synchronize movements to a visual rhythm results in higher variability and reduced timing accuracy (Elliott et al., 2010). However, more recently it has been found that visual cues which present temporal-spatial information rather than just temporal information, improves synchronization performance (Hove et al., 2013b). Synchronizing to multisensory cues results in improved performance in terms of reduced variability in the movement timing statistics (Elliott et al., 2010). Multisensory cues have been shown to improve accuracy in many tasks, ranging from determining object size (Ernst and Banks, 2002) through to the location of where the event occurred (Alais and Burr, 2004). The commonly observed multisensory advantage stems from the brain combining information about an event or object from all available sensory modalities. By weighting each sensory cue according to their relative reliability, a statistically optimal estimate of the event or object can be achieved, explained mathematically by Maximum Likelihood Estimation (MLE; Ernst and Banks, 2002). In a finger-tapping task, presenting participants with a multisensory metronome resulted in reduced asynchrony variability (Elliott et al., 2010). An MLE model was able to accurately predict the reduced variability in bimodal conditions, based on the standard deviation of asynchronies observed when tapping to the associated unimodal metronomes. Here, we investigate whether bimodal presentations of auditory and visual metronome cues can also improve lower limb timing accuracy in the form of stepping in place, relative to their unimodal counterparts.

The effect of rhythmic cueing on lower limb synchronization has tended to be limited to clinical studies. There is strong evidence that rhythmic auditory cueing is effective in the rehabilitation and retraining of gait in both Stroke (Thaut et al., 1997, 2007) and Parkinson’s disease (PD) patients (Nieuwboer et al., 2007; Rochester et al., 2009; Ford et al., 2010), and early evidence that rhythmic auditory cueing may benefit gait in those with Cerebral Palsy (Kim et al., 2012) and Multiple Sclerosis (Conklyn et al., 2010). Subsequent PD patient investigations have shown more limited effects of visual rhythmic cues (Arias and Cudeiro, 2008), with participants showing a strong preference for an auditory cue over a visual cue (Nieuwboer et al., 2007), possibly due to increased attentional demands of a visual cue interfering with gait in this patient population (Rochester et al., 2007). In a healthy population, it is suggested that projected visual light targets onto a treadmill belt outperform discrete auditory metronome cues in influencing the timing of gait (Bank et al., 2011), again likely due to the spatio-temporal cue structure as observed in upper limb synchronization tasks. Multisensory research involving lower limb timing is limited. Using a healthy population, stride interval variability in walking was indicated to be lower for trimodal (auditory, visual and tactile) metronome cue presentations compared to unimodal visual and tactile cues, but not unimodal auditory cues (Sejdić et al., 2012). It is therefore currently unclear how metronome cues presented in different modalities affects lower limb timing in terms of synchronization and correction.

In this study, we investigate both step interval timing and synchronization performance when moving in time to metronomic beats presented in auditory, visual and combined auditory-visual (AV) modalities. Furthermore, we investigate how cue modality affects the temporal correction of movements such that synchrony is maintained, using the phase perturbation paradigm (Repp, 2001, 2002; Elliott et al., 2009a). We predicted that synchronization of steps to the stimuli would be most accurate, in terms of low variability, when participants were presented with bimodal audio-visual cues (Elliott et al., 2010). We further predicted that the increased temporal reliability of the bimodal cues would result in larger step corrections following the phase perturbation. Hence, we expected that participants would regain synchrony with the metronome quicker for the bimodal cues compared to the unimodal presentations.

## Methods

### Participants

Ten participants (Eight female, age range 20–33 years) gave written informed consent to take part in the study. All participants reported themselves free of any neurological disease, head trauma, musculoskeletal impairment, visual impairment or hearing impairment. Ethical approval was granted by the University of Birmingham Science, Technology, Engineering and Mathematics Ethical Review Committee.

### Data collection

All testing occurred during a single session and consisted of cued stepping in place. Participants stepped on two force plates (Bertec 4060H, Ohio, USA), placed side by side, with one foot on each force plate. Participants were instructed to time their foot strikes to the metronome.

The metronome stimuli were presented using the MatTAP toolbox (Elliott et al., 2009b) with a period of 500 ms (cue duration 30 ms). The auditory tones were produced using a piezoelectric buzzer with a tone frequency of 800 Hz. The visual stimuli were produced using a single row array of six green, 5 mm diameter, LEDs. The LED array and buzzer were positioned together at head height, 2 m away from the participant. Each trial consisted of 30 metronome beats presented in auditory-only (AO), visual-only (VO) or simultaneous AV modalities.

**Figure 1:**
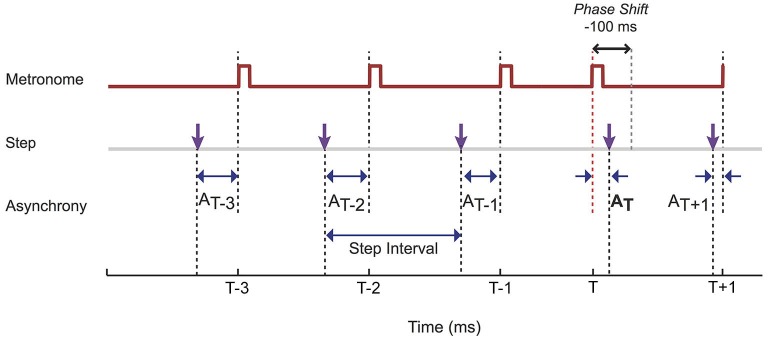
**Steps onsets, defined as the foot strikes registered on the force plates, were compared to the metronome onsets**. The metronome was subjected to a phase shift at time T. Producing this phase shift was achieved by either increasing or decreasing (as illustrated) the duration of the interval preceding T by 100 ms. The step interval is determined by the time between successive step onsets, while the temporal difference between the cue onset and the participant’s step onset is quantified by the asynchrony (*A*). As illustrated, and consistent with previous studies, participants’ movement onsets tended to precede the metronome.

A single phase perturbation was introduced in each trial. Phase perturbations were programmed to occur randomly between the 11th and 18th beat of each trial. This induced a shift of the beat so it occurred either 100 ms earlier (a negative shift) or 100 ms later (a positive shift) than expected. All subsequent beats were shifted by the same amount, such that participants had to correct the timing of their steps to get back in phase with the beat (Figure 1).

Overall, participants completed eight trials for each metronome modality condition, four with a positive phase shift and four with a negative phase shift. A blocked design was used so that each participant received a randomized order of the experimental conditions.

### Data analysis

Data were sampled at 2000 Hz, and filtered using a 2nd order, multi-pass Butterworth filter with a cut-off frequency of 15 Hz. Step timings were determined from initial foot contact on the force plates using a custom analysis script in MATLAB (version 2012a; MathWorks, Natick, MA, USA).

The asynchrony of a foot strike to the metronome beat was determined by subtracting the time the metronome beat occurred from the time the foot strike occurred. Therefore, a positive value indicated the foot strike was behind the metronome (i.e., late) whereas a negative value indicated the foot strike was ahead of the metronome (i.e., early). Left and right foot strike times were merged into a single series. On each trial, “T” is used to describe the foot strike where the phase shift occurred, with “T−1” representing the foot strike immediately prior to the phase shift and “T+1” representing the foot strike immediately after the phase shift, and so on (Figure 1). The accuracy of synchronization was calculated from the mean and standard deviations of the asynchrony measurements in the 10 steps prior to T. Mean and standard deviations of step time intervals (time between consecutive steps) were also calculated using the same 10 steps prior to T.

To assess recovery following the phase shift, the relative asynchrony between each step and metronome beat was calculated. Relative asynchrony was defined as the change in asynchrony following the phase shift perturbation in the metronome. The baseline asynchrony was calculated as the mean of the asynchronies at T−5 through to T−1 and was subsequently subtracted from the remaining asynchronies from beat T onwards, to get the relative asynchronies (Elliott et al., 2009a). To quantify the rate of recovery from the phase perturbation, we calculated the percentage correction to the relative asynchrony at T, attributed to each subsequent step from T+1 to T+10.

### Statistical analyses

The mean and SD of the asynchronies and the step intervals were analyzed using a Repeated-Measures ANOVA based on a 3 (Modality: AO, VO, AV) × 2 (Perturbation: Positive, Negative) design. Phase perturbation recovery was similarly analyzed using a Repeated-Measures ANOVA based on a 3 (Modality: AO, VO, AV) × 2 (Perturbation: Positive, Negative) × 5 (Step: T+1 through to T+5) design. Statistical analyses were implemented using SPSS (v21; IBM Corp., New York, US). Sphericity violations were corrected for using Greenhouse-Geisser adjustments. *Post hoc* analysis *p*-values were adjusted using the Bonferroni method. Results were considered significant for *p* < 0.05.

## Results

Using metronomes presented in AO, VO and combined AV modalities, we investigated participants’ ability to step in time to the beat. Each trial consisted of 30 metronome beats with a regular interval of 500 ms. In addition, we phase perturbed a single beat in each trial such that it occurred earlier or later than expected. Subsequent beats remained at the shifted phase, which meant participants had to correct the timing of their steps to get back in time with the beat.

**Figure 2:**
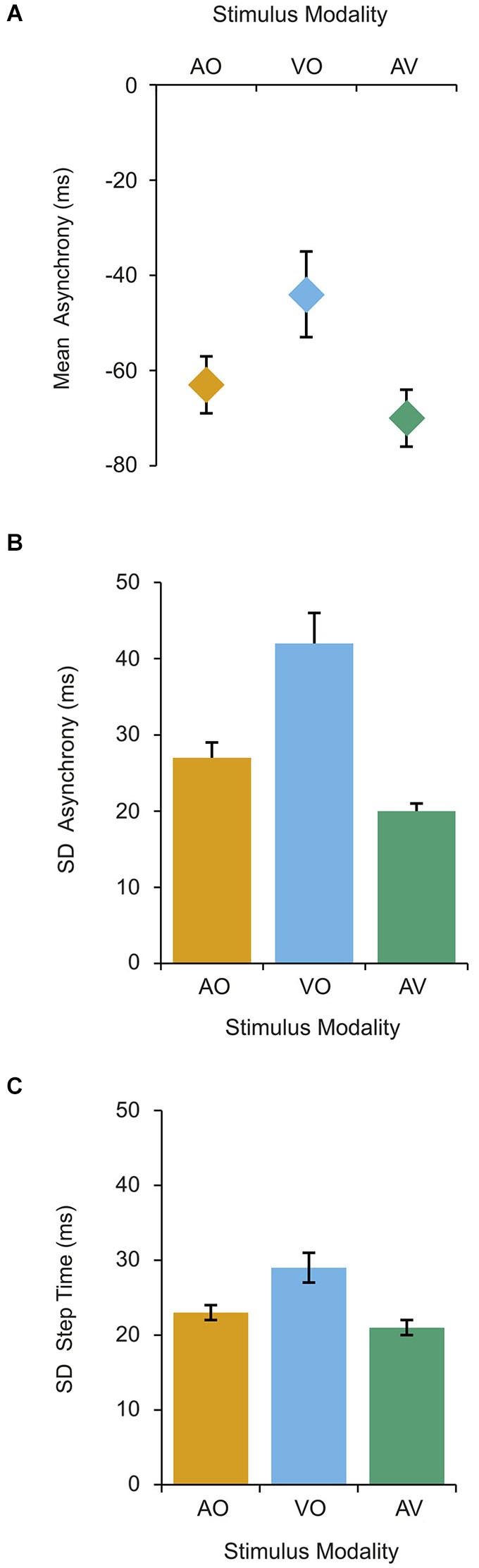
**Asynchronies and step-intervals were calculated for the ten steps prior to the phase perturbation**. For each condition, we calculated **(A)** the mean asynchrony, **(B)** the standard deviation of asynchrony across the ten steps. **(C)** We further calculated the standard deviation of the step-intervals over the ten steps. Error bars represent standard error of the mean (SEM).

We found that the sensory modality of the metronome beats affected the participants’ ability to synchronize their steps in terms of mean (*F*_(2,18)_ = 7.68, *p* = 0.004) and variability (s.d.) of the asynchronies (*F*_(2,18)_ = 21.81, *p* < 0.001). As reported in many sensorimotor synchronization studies (Repp, 2005), we observed a negative mean asynchrony for all conditions (Figure 2A). However, we found that participants stepped slightly later in the VO condition relative to the AV condition (*p* = 0.034), whereas there was no notable difference between the AV and AO modalities. Importantly, we further observed that asynchrony variability was higher in both single modality conditions, relative to the AV condition (AO, *p* = 0.004; VO, *p* = 0.001; Figure 2B), highlighting that timing accuracy of the steps was improved with the bimodal metronome cues. As predicted, the visual metronome resulted in the highest asynchrony variability (*p* = 0.015, relative to AO).

Step time variability was also affected by modality (*F*_(2,18)_ = 13.36, *p* < 0.001; Figure 2C) with participants producing more variable step intervals when synchronizing with the visual cue (relative to AO, *p* = 0.005; AV, *p* = 0.002). However, between the AO and AV conditions there was no difference in step time variability. Furthermore, participants’ mean step time was not affected by the modality of the metronome cues, and accurately matched the metronome interval (*M* = 497 ms, S.E = 10 ms; averaged across all three modality conditions).

**Figure 3:**
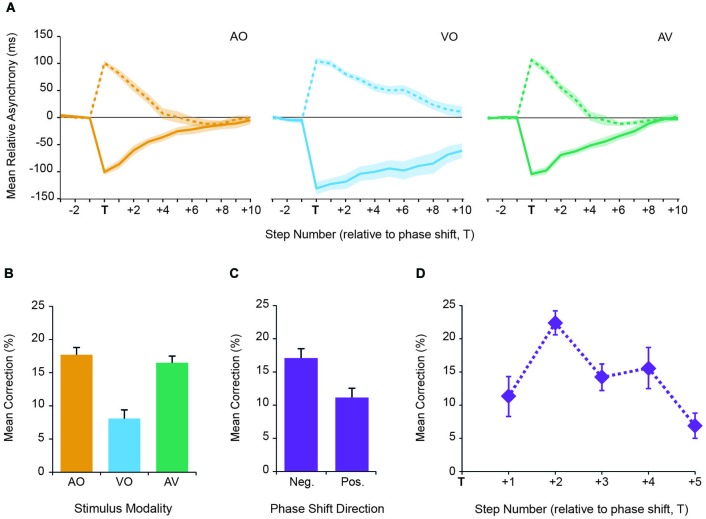
**(A)** Relative asynchrony between the step onset and the metronome onset for the three sensory modalities. Onset T corresponds to the occurrence of the phase perturbation. Zero relative asynchrony is defined as the mean asynchrony between time points T−5 and T−1. Responses to a positive phase shift (+100 ms; solid line) and a negative shift (−100 ms; dashed line) are shown, with data points representing the between subjects means. Error bars represent SEM. **(B)** Mean percentage correction for each sensory modality. **(C)** Mean percentage correction for positive and negative phase shifts. **(D)** Mean percentage correction for steps following the perturbation (T+1 to T+5). Mean correction was defined as the percentage change in relative asynchrony per step, with respect to the relative asynchrony at the point of the perturbation (step T). Data represent the between subjects mean. Error bars represent SEM.

Following the phase perturbation, we investigated how quickly participants corrected the timing of their steps to get back into synchrony with the metronome. The phase of the metronome was always shifted by the same amount (100 ms), but was either a negative shift (the beat was shifted earlier) or positive (the beat was shifted later). The phase perturbation occurred on a random beat between beats 11 and 18. We analyzed the correction response to the phase shift by measuring the mean relative asynchrony between steps and the metronome (see Methods). We observed that participants responded to the phase shift and attempted to resynchronize with the beat across all conditions (Figure 3A). However, responses differed across conditions. To investigate how the conditions affected how participants corrected their steps, we analyzed the percentage correction on each step, relative to the initial phase shift asynchrony (step T) for the subsequent five steps. We found that metronome modality affected step correction (*F*_(2,18)_ = 40.76, *p* < 0.001; Figure 3B). More specifically, the mean percentage step correction for the visual metronome was significantly smaller than for either auditory or AV metronomes (*p* < 0.001). In contrast, there was no significant difference between auditory and AV metronomes. There was also a difference in the percentage correction depending on the phase shift direction (*F*_(1,9)_ = 8.67, *p* = 0.016; Figure 3C), with greater correction following a negative shift compared to a positive shift. We observed no interactions between these factors. Finally, we found that the percentage correction changed relative to the number of steps after the phase shift (*F*_(2.19, 19.67)_ = 5.125, *p* = 0.014; Figure 3D). In particular, it appeared that participants made the largest correction on the second step. As participants got closer to their target phase, their corrections reduced, with the 5th step having a significantly lower percentage correction relative to the 2nd step (*p* = 0.024).

## Discussion

In the present study, we investigated the effects of cue modality (auditory, visual, and combined auditory-visual) on lower limb synchronization to sequences of rhythmic events, which were subjected to phase perturbations occurring at unpredictable times. It was hypothesized that multisensory cues would facilitate synchronization of lower limb movements more than when the same cues were presented unimodally. The participants displayed lower variability in their asynchrony in the combined AV condition compared to both the AO and VO cues. The recovery from a phase perturbation was not significantly different between the AO and AV cues. However, recovery in these conditions was superior to VO cueing. Similarly, there was no significant difference in the step interval variability between AO and AV conditions, although step variability in the VO condition was substantially higher.

Cueing with an auditory component has previously been shown to reduce variability more than for a visual cueing condition during treadmill walking (Sejdić et al., 2012), and is suggested to be due to rhythmic entrainment to the auditory component of the stimuli resulting in more consistent motor recruitment patterns (Miller et al., 1996). Neuroimaging studies have shown that listening to an auditory rhythm engages motor areas in the brain, even in the absence of any movement itself (Grahn and Brett, 2007). A meta-analysis of imaging studies involving upper limb movement reveals that the brain regions activated using a visual pacing stimulus are distinct from the regions activated using either an auditory stimulus or when no pacing is present (Witt et al., 2008). Furthermore, activity in the putamen is highest for a discrete auditory signal compared to a continuous auditory cue or a discrete or continuous visual cue (Hove et al., 2013a). As the putamen is a key area for sensorimotor synchronization stability (Hove et al., 2013a), the increased activity during auditory as opposed to visual cueing may account for the reduced variability in the AO compared to the VO conditions observed in this study and previous finger tapping studies.

For the condition where both auditory and visual cues were presented (AV), we saw a further reduction in asynchrony variability compared to both the AO and VO conditions. This indicates that participants weren’t ignoring the visual cue in favor of the more reliable auditory cue, but in fact integrating the two sensory sources together to get a better estimate. This method of integration can be shown to be the statistically optimal way of estimating cue properties and has been found to be present across a range of perception tasks (Ernst and Banks, 2002; Alais and Burr, 2004; Elliott et al., 2010). In particular, it was found that when tapping along to multisensory cues, which were manipulated in their temporal reliability, participants integrated the sensory information weighting more toward the most reliable cue (Elliott et al., 2010). Described mathematically by MLE, it was shown the resulting estimates of the beat onsets were optimal and this subsequently resulted in participants reducing their asynchrony variability compared to tapping along to beats in the associated single modality conditions. Here, we observed a similar result in the variability of the step asynchronies. Hence, we have replicated the effect of integrating rhythmic sensory cues in the task of lower limb synchronization, highlighting that multisensory cues can improve temporal accuracy of motor responses, regardless of effector.

Step interval variability was not found to be significantly lower in the presence of bimodal cues compared to auditory only. The step interval variability quantifies the regularity of the step-to-step timing, in contrast to the asynchrony variability determining the regularity of the step-metronome timings. While we found the asynchrony variability showed a strong multisensory reduction, we did not see the same for the step interval variability. This suggests that participants were indeed performing the task of synchronizing as best they can to the metronome beat, as instructed. That is, they were minimizing the asynchrony variability of their steps to the metronome. To achieve this, continuous correction is required to remain in phase with the beat, which can subsequently lead to increased step interval variability (Vorberg and Schulze, 2002). In contrast, if one were to minimize step interval variability, phase correction to the metronome would also be reduced and lead to accumulating errors and higher asynchrony variability.

Correction to the phase shift was slowest in the VO condition regardless of the direction of the phase shift. We suggest that the increased uncertainty in the registration of the visual cues subsequently reduces the level of correction each participant makes on each step, to regain synchrony with the visual beat. A reduction in phase correction performance has previously been shown when the sensory feedback from the movement itself (measured in terms of mean squared jerk or yank) is unreliable (Elliott et al., 2009a). Here, we show that a similar effect occurs when it is the external stimulus, not one’s own movement that has the uncertainty. This is an intuitive result: if the central nervous system has reliable information about the error between our own movement and that of the external cue, then the error should be corrected as quickly as possible. However, if there is high uncertainty about the the error (due to an unreliable estimate of the cue onset or our own movement) then the magnitude of the correction should be limited. This process of correction also appears to be related to high a cost of over-correction. The participants in this study timed their foot strikes slightly ahead of the metronome beat in all cue modalities. This is consistent with finger tapping studies (Wing et al., 2010), as well as lower limb responses such as foot tapping during sitting and standing (Chen et al., 2006) and some gait studies (Pelton et al., 2010; Roerdink et al., 2011). This anticipatory response is still not fully explained in the literature, but suggests an associated cost with timing movements to occur after the beat has occurred. Moreover, we noted that regardless of modality, participants corrected their movements faster to negative phase-shifts (resulting in positive asynchronies) than to positive phase-shifts (resulting in an increased negative asynchrony). This again points to an increased cost of the participant timing their movement late, rather than early, relative to the beat.

Finally, we found that despite reduced asynchrony variability in the AV condition relative to the unimodal conditions, the percentage correction following the phase shift did not significantly differ between AO and AV. While this appears to go against our suggestion that correction increases with cue reliability, we suggest the level of correction may become limited by motor factors when reliability of the cues is high. That is, prioritizing postural stability in the stepping process may become the dominating factor over producing large temporal corrections that could incur instability (Brauer et al., 2002; Pelton et al., 2010; Liston et al., 2014). This is further evidenced by the smaller timing corrections overall, compared to the reported correction values to perturbations presented in finger tapping. While we found average step-by-step corrections of between 10–20% (across sensory modalities, Figure 3D), corrections in similar finger tapping studies have typically been reported to be in the region of 50% to an auditory metronome (Repp, 2001, 2008) and 30% to a visual metronome (Repp and Penel, 2002). Hence, a more complex movement task (i.e., maintaining postural stability) appears likely to impact on the magnitude of the correction response to a phase perturbation.

It is useful to consider how our results are relevant to the applications of movement rehabilitation in neurological diseases. Stepping in place to an auditory cue has been shown to immediately reduce the excessive step time variability observed in hemiparetic stroke (Wright et al., 2013), and walking to an auditory cue reduced excessive stride time variability in individuals with PD (Hausdorff et al., 2007). While our results indicated that step time variability was not reduced in AV compared to AO modalities, the fact the asynchrony variability was reduced in AV conditions suggests multisensory cues can encourage improved synchronization to external cues. Therefore, multisensory presentations could be used to encourage greater levels of temporal correction in lower limb movements and hence re-train adaptive gait in the aforementioned pathological populations.

In conclusion, we have demonstrated that multimodal audio-visual metronome cues results in greater stability of sensorimotor synchronization during stepping in place compared to unimodal AO or VO cues. Our results are consistent with previous findings in upper limb tasks such as finger tapping, suggesting a common mechanism for temporal processing of sensory information, regardless of effector.

## Authors and contributions

RLW and MTE designed the experiment. LCS was responsible for running the experiment and data processing. MTE and RLW conducted the data analysis and wrote the manuscript.

## Conflict of interest statement

The authors declare that the research was conducted in the absence of any commercial or financial relationships that could be construed as a potential conflict of interest.
