# Corrigendum: Stepping to phase-perturbed metronome cues: multisensory advantage in movement synchrony but not correction

**DOI:** 10.3389/fnhum.2015.00441

**Published:** 2015-08-19

**Authors:** Rachel L. Wright, Laura C. Spurgeon, Mark T. Elliott

**Affiliations:** School of Psychology, College of Life and Environmental Sciences, University of BirminghamEdgbaston, Birmingham, UK

**Keywords:** stepping, sensorimotor synchronization, multisensory integration, corrigendum, gait timing

Due to an oversight, one of the co-author's names was left out of the original article. The list of authors in the original article has now been updated to include Laura C. Spurgeon who made a significant contribution to this study, including its organization and conduct, as well as some aspects of the design.

The Author Contributions section of the original article were also updated to include Laura C. Spurgeon and the Acknowledgments section was further amended as necessary.

The authors regret the earlier omission. This error does not change the scientific conclusions of the article in any way.

The original article has been updated.

## Conflict of interest statement

The authors declare that the research was conducted in the absence of any commercial or financial relationships that could be construed as a potential conflict of interest.

